# Motor and default mode network states of rest in frontal lobe epilepsy

**DOI:** 10.1016/j.ynirp.2025.100278

**Published:** 2025-06-28

**Authors:** Tahereh Rashnavadi, Raphael F. Casseb, Kristine E. Woodward, Paolo Federico, Bradley Goodyear

**Affiliations:** a Department of Biomedical Engineering, University of Calgary, Canada; b Department of Neurology, University of Campinas, Brazil; c Department of Pediatrics, Section of Neurology, University of Calgary, Cumming School of Medicine, Alberta Children's Hospital, Calgary, Canada; d Hotchkiss Brain Institute, Cumming School of Medicine, University of Calgary, Canada; e Seaman Family MR Research Centre, Cumming School of Medicine, University of Calgary, Canada; f Department of Clinical Neurosciences, Cumming School of Medicine, University of Calgary, Canada; g Department of Radiology, Cumming School of Medicine, University of Calgary, Canada

## Abstract

Frontal lobe epilepsy (FLE), marked by recurrent seizures arising from the frontal lobes, can significantly impair cognitive and motor function, reducing quality of life. Recent studies suggest that epilepsies can involve functional networks throughout the brain that can be identified using resting-state functional magnetic resonance imaging (fMRI). In this study, we aimed to determine whether FLE is associated with a distinct functional network brain states. Using dynamic functional connectivity analysis in combination with *k*-means clustering, we investigated dynamic connectivity patterns of the somatomotor network (SMN) and default mode network (DMN) of ten right-hemisphere and six left-hemisphere FLE patients, as well as nine healthy controls. We found two distinct states of rest for both the SMN and DMN: a high connectivity state and a lower, more variable connectivity state that was often specific to individual patients. Both FLE groups showed reduced overall connectivity compared to controls, with the greatest differences emerging during the low connectivity state. Right FLE patients and controls exhibited relatively uniform reductions, whereas left FLE patients showed spatially specific disruptions, including reduced lateral-to-medial SMN connectivity and decreased connectivity in posterior and left-lateralized DMN regions. Our findings suggest that dynamic connectivity analysis can uncover the temporal complexity and patient-specific nature of brain network disruption in FLE, supporting the development of personalized diagnostic and treatment strategies. Further research with larger cohorts is necessary to validate these results and explore additional factors affecting brain functional connectivity.

## Highlights

• Identified two distinct resting states in the somatomotor and default mode networks in frontal lobe epilepsy patients.
• Demonstrated the utility of dynamic functional connectivity analysis combined with *k*-means clustering in a small clinical population.
• Highlighted the variability of connectivity states specific to individual patients, suggesting implications for personalized diagnostics.
• Provided insights into the temporal complexity of brain functional connectivity in frontal lobe epilepsy.

## 1 Introduction

Frontal lobe epilepsy (FLE) is the second most common type of epilepsy (Englot et al., 2012) and is characterized by recurrent seizures that originate within the frontal lobes. Individuals with FLE can suffer from a number of cognitive and behavioral symptoms, including difficulties with attention, memory, and executive function, as well as motor deficits such as impaired coordination, decreased dexterity, poor motor planning skills, amongst others (McGonigal, 2022; Arrotta et al., 2022). As a result, FLE can have a negative impact on the performance of daily tasks, significantly reducing quality of life. While FLE can be adequately treated using anti-seizure medications, many cases are drug-resistant and require surgery. Unfortunately, the success rate of surgical resection is highly variable, ranging from 13% to 80% (Jeha et al., 2007). Inaccurate localization of seizure-generating tissue and, thus, its incomplete resection remain major clinical issues (West et al., 2019). In addition, the impact of focal epilepsies such as FLE may extend to larger functional networks, either directly through seizure propagation or indirectly via brain regions that bridge networks (Laufs, 2012; Robinson et al., 2017; Royer et al., 2022).

Recently, resting-state functional magnetic resonance imaging (fMRI) has become an important tool to help understand epileptic networks throughout the brain. By examining the temporal synchrony of fMRI signals, one can determine networks of brain regions whose synchronous activities may be the result (or the source) of aberrant seizure activity (Abbott et al., 2019). In healthy individuals, synchronous activity between brain regions is thought to define functional networks, where the degree of synchrony (termed connectivity) is believed to reflect the strength of a functional connection. Thus, resting-state fMRI also has the potential to identify how epilepsy impacts the connectivity of existing brain networks, which could improve our understanding of the symptoms experienced by individuals with epilepsy.

Most resting-state fMRI studies focused on temporal lobe epilepsy (Tracy and Doucet, 2015; Pereira et al., 2010; Waites et al., 2006) due to its prevalence. Of the limited number of resting-state fMRI studies of FLE, one study from our group demonstrated reduced interhemispheric connectivity between the somatomotor cortices proportional to the number of lifetime seizures (Woodward et al., 2014), suggesting a direct impact of repeated seizure activity on the organization of the somatomotor network (SMN) in FLE patients. The SMN, which encompasses the primary motor and somatosensory cortices, supplementary motor areas, and relevant subcortical regions, is particularly pertinent, given the frequent involvement of motor areas in seizure onset and propagation. Motor deficits are common in FLE and have been associated with altered connectivity within the SMN; however, few studies have explicitly examined this network through the lens of dynamic functional connectivity (Klugah-Brown et al., 2019; Li et al., 2024).

Regardless of the type of epilepsy, resting-state fMRI studies have investigated connectivity of the default mode network (DMN). The DMN is a large-scale network of brain regions, including the dorsal medial prefrontal cortex, posterior cingulate cortex and angular gyrus, that is believed to be primarily active during cognitive processes such as self-focus, planning and remembering of events and less active during cognitively demanding tasks that require focus. Interestingly, connectivity within the DMN has been shown to be modulated by several neurological conditions, including epilepsy (Lopes et al., 2014; Morgan et al., 2012; Laufs, 2012; Waites et al., 2006; Zhang et al., 2010a, Zhang et al., 2010b; Liao et al., 2010; Pittau et al., 2011; McCormick et al., 2013; Laufs et al., 2007; Fahoum et al., 2012; Broyd et al., 2009). Although altered connectivity of the DMN does not appear to be disease-specific, understanding when changes in connectivity of the DMN occur may help to identify seizure-generating regions within the DMN or elsewhere. This will require an approach to connectivity analysis that can determine when connectivity of the DMN changes, not just where. While typical resting-state fMRI analyses determine the average connectivity over a 5- to 10-min span, there are fluctuations in connectivity that occur on the order of seconds that are believed to indicate interactions between networks (Chang and Glover, 2010); that is, connectivity is time-varying or dynamic. The dynamic nature of connectivity is a particularly important consideration for epilepsy, given that some form of change in connectivity is likely taking place at the onset or during seizures or possibly in between seizures (interictally). Hence, a reliable method to examine changes in connectivity over time is needed to successfully identify brain networks associated with epilepsy and, further, if these networks define a temporally stable state of the brain.

Our group has developed a dynamic connectivity analysis approach based on hierarchical observational modeling (Sojoudi and Goodyear, 2016). This approach can determine when connectivity significantly changes at any moment in time. Its framework consists of a two-level linear model composed of overlapping sliding windows of fMRI signals, incorporating the fact that overlapping windows are not independent; it also includes a model of noise for each window. This technique possesses greater sensitivity and specificity for detecting regions of variable connectivity compared to sliding-window approaches based on temporal cross-correlation analysis. Estimates of connectivity over time between brain regions form a time series of connectivity matrices that can be examined using *k*-means clustering to identify *k* connectivity patterns (i.e., brain states) that are temporarily stable during a resting-state fMRI acquisition. Using this approach and given the established impact of FLE on the SMN and DMN, we hypothesized that the SMN and DMN each exhibit a state of rest in FLE patients that differs from that of healthy controls. Analyzing these networks separately allowed us to isolate network-specific dynamic alterations that may otherwise be missed in whole-brain analyses. This targeted approach also mitigated overfitting risks associated with high-dimensional data clustering in small clinical samples. Moreover, the use of the hemispherically-resolved multiresolution brain atlas (MIST; Urchs et al., 2019), which provided both right and left hemisphere representations of regions in the SMN and DMN, enabled precise characterization of lateralized abnormalities in these functionally distinct networks.

Understanding dynamic connectivity within the SMN and DMN could offer a more complete view of how FLE disrupts brain function. Prior studies have linked disruptions in frontoparietal and motor networks to seizure onset age and cognitive performance in FLE (Widjaja et al., 2013; Klugah-Brown et al., 2019; Li et al., 2024), underscoring the importance of focusing on clinically relevant networks. Identifying distinct dynamic states within these networks may provide insights into compensatory or maladaptive processes related to seizure burden, cognitive dysfunction, or treatment response (Liao et al., 2010; Zhang et al., 2010a; Yang et al., 2021). By characterizing brain state dynamics that may be obscured by static connectivity analyses, this study aims to contribute to the development of connectivity-based biomarkers to inform prognosis and support personalized epilepsy care.

## 2 Methods

### 2.1 Participants

This study was approved by the Conjoint Health Research Ethics Board of the University of Calgary. Written informed consent was obtained from all participants prior to participation. Ten patients with right-hemisphere FLE (2 female; age = 35.5±13.1 years) and six with left-hemisphere FLE (2 female; age = 39.3±17.5 years) were recruited through the University of Calgary Comprehensive Epilepsy Program. FLE was confirmed by history, examination, routine electroencephalography (EEG), video-EEG monitoring, and/or anatomical MRI, PET and SPECT. Exclusion criteria included previous neurosurgery and contraindications to MR imaging, which were established through a pre-screening questionnaire. Nine healthy controls (5 female; age = 29.9±13.0), with no known, reported neurological or psychiatric disorders, were recruited through word of mouth. All participants were right-handed. Patient details are given in Table 1.

**Table 1:** Clinical information of left (L) and right (R) frontal lobe epilepsy patients. Seizure types are listed as: focal aware seizures (FAS), focal impaired awareness seizures (FIAS), or focal to bilateral tonic-clonic seizures (FBTCS). Seizure burden is based on the time between seizures: low (> 6 months), moderate (1–6 months), high (2–4 weeks), or very high (< 2 weeks).

PARTICIPANT	SEX	AGE (YEARS)	AGE OF ONSET (YEARS)	SEIZURE FOCUS (BURDEN)	SEIZURE TYPES
**1L**	M	30	8	Primary motor (High)	FAS, FBTCS
**2L**	M	28	2	Primary motor (Very high)	FIAS, FAS, FBTCS
**3L**	F	65	41	Primary motor (Low)	FIAS, FAS
**4L**	F	55	12	Primary motor (Low)	FAS, FBTCS
**5L**	M	19	4	Supplementary sensorimotor (Low)	FIAS, FAS, FBTCS
**6L**	M	39	2	Dorsolateral (Very high)	FIAS, FBTCS
**1R**	M	16	7	Supplementary sesnsorimotor (Low)	FIAS, FBTCS
**2R**	M	48	28	Supplementary sensorimotor (Low)	Nocturnal FBTCS (3 in total)
**3R**	M	33	24	Right orbitofrontal (Moderate)	FBTCS (0.5/yr), FAS (1/month)
**4R**	M	18	12	Right posterior frontal (Low)	FBTCS (1–2/year), FAS (1/year)
**5R**	M	59	<0.2	Right sensorimotor (Very high)	FBTCS (none now), FAS (3–4/week), FAS (2–3/year)
**6R**	F	34	5	Right frontal and temporal (Very high)	FAS (1–2/day), Drop (2–3/month), FBTCS (1 every 3–4 months)
**7R**	M	33	28	Anterior frontopolar (Low)	FIAS
**8R**	F	36	0.4	Right parasagittal frontal (Low)	FBTCS (1/4 months)
**9R**	M	32	26	Anterior frontopolar (Moderate)	FAS
**10R**	M	46	39	Anterior frontopolar (Low)	FBTCS

### 2.2 MRI data collection and processing

MRI data were acquired using a 3.0 T Discovery MR750 whole-body scanner with a receive-only 8-channel phased-array head coil (GE Healthcare, Waukesha, WI). Resting-state fMRI data with blood oxygen level-dependent (BOLD) contrast were collected using a gradient-recalled echo, echo planar imaging sequence (150 vol (5 min), voxel dimensions 3.75 × 3.75 × 4 mm, 28 slices, 64 × 64 matrix, TE/TR = 30/2000 ms, flip angle = 65°). A 3D magnetization-prepared gradient-echo sequence (2-mm slices, 384 × 256 × 112 matrix, preparation time = 500 ms, minimum TE, TR = 8.9 msec, flip angle = 20°) was used to collect T1-weighted images for anatomical registration of the fMRI data and to localize regions of interest.

Prior to analysis, resting-state fMRI data underwent several processing steps using the *FMRIB Software Library* (FSL, http://www.fmrib.ox.ac.uk/fsl), including slice timing correction, spatial smoothing using a Gaussian kernel with a full-width at half-maximum of 6 mm, high-pass temporal filtering (Gaussian-weighted least-squares straight line fitting, with sigma = 50.0 s), and non-brain removal using the *Brain Extraction Tool* (BET) (Smith, 2002). Motion correction was carried out using the *Motion-Correction FMRIB Linear Image Registration Tool* (MCFLIRT) (Jenkinson et al., 2002). FSL's *Multivariate Exploratory Linear Optimized Decomposition into Independent Components* (MELODIC) tool was used to decompose each resting-state fMRI dataset into independent component maps of spatial clusters exhibiting a similar temporal signal. All maps were visually inspected to identify components attributed to noise or artifacts according to previously described criteria (Griffanti et al., 2017). The temporal signals of these components were then removed from the resting-state fMRI data by linear regression. Finally, the resting-state fMRI data were registered to the anatomical images using *FMRIB Linear Image Registration Tool* (FLIRT) (Jenkinson et al., 2002; Jenkinson and Smith, 2001). The adverse effects of global signal regression during preprocessing remain a topic of debate (Murphy et al., 2009; Power et al., 2017); therefore, global signal regression was not included as a preprocessing step.

Anatomical images were then registered to a multiresolution anatomical/functional brain atlas (MIST; Urchs et al., 2019); 12 regions of interest (ROIs) were segmented across both hemispheres from the SMN (see Supplementary Table 1), and 38 ROIs were segmented from the DMN (see Supplementary Table 2). For each ROI, the mean resting-state fMRI time course was extracted, and connectivity as a function of time was computed between all possible ROI pairs within each network using a sliding window approach based on hierarchical observational modelling (Sojoudi and Goodyear, 2016). Specifically, connectivity was calculated for 100-s segments of data that shifted one volume at a time, resulting in 140 estimates of connectivity over the duration of the fMRI acquisition. This yielded 140 connectivity matrices of size 12 × 12 for the SMN and 38 × 38 for the DMN, per participant.

### 2.3 Group-based analysis

The following analyses were conducted for each of the SMN and DMN separately. For each participant group (controls, left FLE, and right FLE), the connectivity matrices were concatenated across participants and underwent *k*-means clustering (with *k* varying from 2 to 5) to classify each connectivity matrix as one of *k* states. To select the value of *k*, the data from each group first underwent four methods that have been shown to optimize the choice of *k* (*k*_*opt*_). These methods were Elbow (Bezdek, 1984), Silhouette (Rousseeuw, 1987), Dunn's score (Dunn, 1973), and Davies-Bouldin (Davies and Bouldin, 1979). Graphical results of these methods were inspected, and *k*_*opt*_ was chosen based on the best agreement among the methods. In cases where agreement was not reached by more than two methods, we also investigated other values of *k* defined by those methods.

For the main analysis with *k* = *k*_*opt*_, *k*-means clustering was performed in MATLAB (MathWorks, MATLAB 2022b) using the *"city block"* (or Manhattan) distance, which separates clusters based on the absolute difference between corresponding elements of the connectivity matrices. This approach has been demonstrated to be highly suitable for high-dimensional data and is robust to outliers (Aggarwal et al., 2001). A total of 200 iterations were performed. This resulted in *k*_*opt*_ connectivity matrices representing *k*_*opt*_ temporarily stationary network states for each participant group. These states were then examined within and between groups by matrix subtraction. The between-group differences in brain states were visualized using brain surface plots generated with Nilearn's plot_connectome (v0.10.0) using predefined MNI coordinates for SMN and DMN regions.

To assess the temporal properties of dynamic states, the percentage of the total scan time spent in each state (state occupancy) was computed for each participant. Graphs of state vs. time were also generated to inspect the transitions between the *k*_*opt*_ states and to assess the frequency and stability of state occurrences over time. Finally, as a means of determining how well each participant was represented by the group states, the sum of squared distances (SSD) between each group state and the corresponding connectivity matrices of each participant was computed, based on their assigned state at each time point. This analysis was then repeated by setting *k* equal to the number of participants in a group to determine if participants were unique in their brain states or if there were common states amongst participants of the group.

To further determine the clinical relevance of our technique with regard to disease severity, we wished to determine if seizure burden had an impact on the determination of brain states, as indicated by the SSD of each participant for each brain state. Due to limited group sizes, seizure burden for each patient was categorized according to two categories: low (low + moderate, as per Table 1) or high (high + very high, as per Table 1), and SSD was tested between seizure burden groups, regardless of left or right FLE, using the Mann-Whitney *U* test.

## 3 Results

### 3.1 Somatomotor network (SMN)

**Fig. 1:**
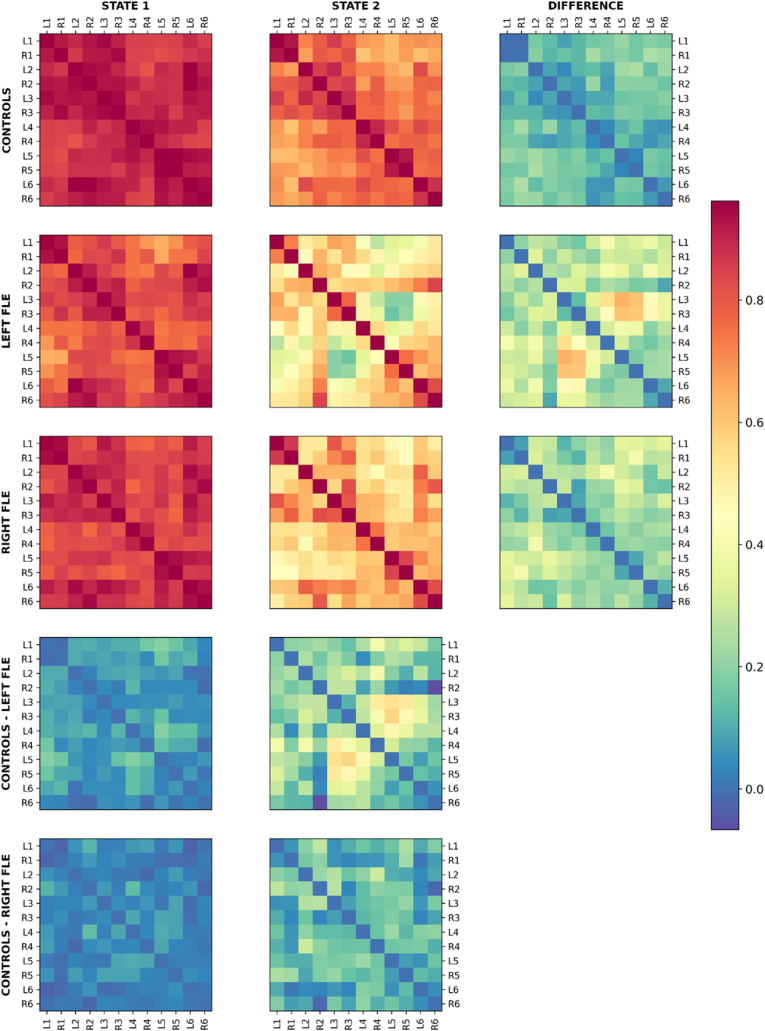
The two connectivity matrix states of the somatomotor network (SMN) (top left and top middle columns) identified by *k*-means clustering for control, left FLE, and right FLE participants. The top right column shows the subtraction of the two states within each group. The bottom two rows depict the subtraction of the states between control and each of the left and right FLE groups. While the control and right FLE groups exhibited a more overall reduction in connectivity in the second state, the left FLE group exhibited a second state with a greater reduction in connectivity between the lateral regions of the SMN (L3/R3) and the anteromedial (L4, R4) and medial regions (L5, R5). Differences between controls and each of the left and right FLE groups for each state are shown in the bottom left and middle. In control and right FLE groups, state differences were marked by a uniform reduction in connectivity across the SMN, whereas the second state in left FLE group showed specific reductions between lateral and anteromedial/medial regions in both hemispheres.

**Fig. 2:**
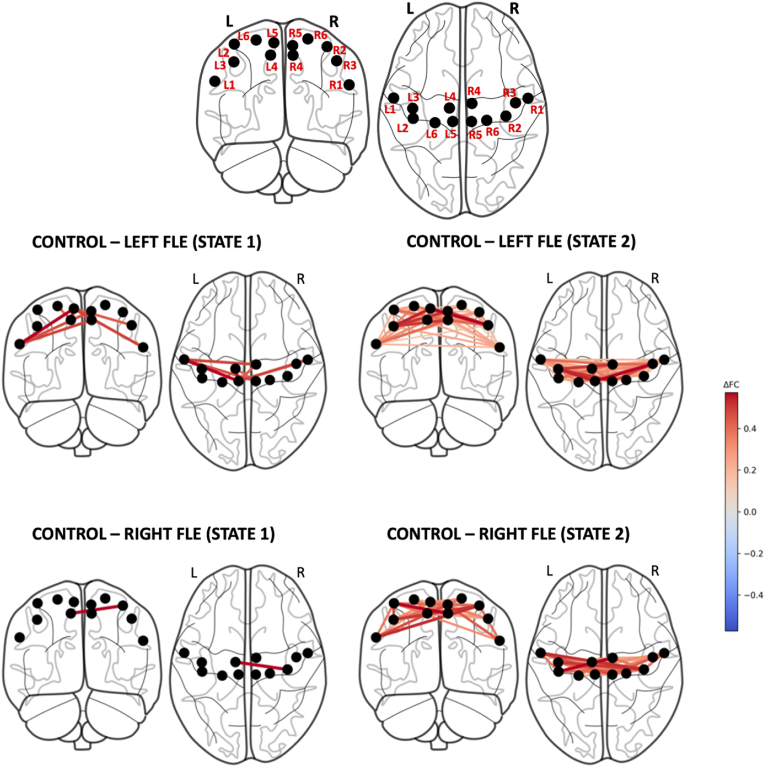
Brain models depicting the differences in connectivity of SMN brain states between controls and left FLE patients (middle row) and right FLE patients (bottom row). For left FLE patients, states were associated with reductions in connectivity of lateral-to-medial connections, with more distributed reductions for State 2. For right FLE patients, relative to left FLE patients, connectivity reductions were less prominent for State 1 and more distributed and greater for State 2. The brain regions are labelled in the top row, as per Supplementary Table S1.

For the SMN, *k = 2* was the consensus between all four clustering validity indices (Elbow, Silhouette, Davies-Bouldin, and Dunn's Score) for all groups (see Supplementary Fig. 1). Fig. 1 shows the two connectivity matrix states for each group, the difference between the two states for each group, and the difference between controls and each of the left and right FLE groups for each state. Regardless of group, one state consisted of high connectivity throughout the SMN, and the other state consisted of lower, more variable connectivity. While the difference between states for the control and right FLE groups was predominantly a uniform reduction in connectivity throughout the SMN, the second state of the left FLE group exhibited regionally specific reductions in connectivity between lateral regions of the SMN and the anteromedial and medial regions in both hemispheres. These findings are also shown using brain surface models in Fig. 2.

State occupancy, SSD, and state transition plots for all participants are provided in Table 2. With the exception of participants 2C and 9C, all control participants occupied both SMN states, with a median occupancy of 54 % for State 1. The SSDs of control participant 6C for each state, however, were considerably higher than the other participants, suggesting that the group SMN states may not be representative of this participant. With the exception of participant 6C, all participants exhibited two or fewer transitions between states, suggesting stable SMN states of rest, confirming low frequency of state transitions. For the left FLE group, five participants occupied State 1 only, while participant 6L occupied State 2 only. This suggests that the two states determined by *k*-means clustering were not actually representative of the group, but instead identified an individual left FLE participant that is unlike the other members of the group. The absence of state transitions in the left FLE group suggests a lack of transitioning, reflecting highly stable and persistent states. For the right FLE group, five out of 10 participants occupied both states, whereas three participants occupied only State 1 and two participants occupied only State 2. Thus, while the states determined by *k*-means clustering for right FLE may be more representative of the group (as indicated by low SSDs across participants), there are differences amongst participants. Overall, a median state occupancy of approximately 43 % for State 1 suggests that right FLE participants actually spent more time in State 2 characterized by lower connectivity. Participants who occupied both states exhibited only one or two transitions, reflecting a low frequency of state transitions. The procedure was repeated for the left FLE group after removing participant 6L to see if *k-*means clustering would identify states more representative of the group. The results are shown in Supplementary Fig. 2. Three of the five participants occupied State 1 only, while one participant (2L) occupied only State 2. One participant (3L) spent 78 % of the time in State 1. State 2 was associated with decreased connectivity between the ventrolateral, anteromedial and medial regions of the SMN in both hemispheres. Hence, *k*-means clustering again identified another left FLE individual who was unlike the other members of the group. For the SMN, seizure burden was not significantly associated with the SSD of either State 1 (U = 28, p = 0.28) or State 2 (U = 13, p = 0.29).

**Table 2:** Somatomotor network state occupancy and sum of squared distances for each participant of each group (C = controls, L = left FLE, R = right FLE), as well as state versus time plots. Median and median absolute deviation (MAD) are reported to summarize central tendency and variability.

PARTICIPANT	STATE 1 OCCUPANCY(%)	SSD TO STATE 1 (× 10^5^)	SSD TO STATE 2 (× 10^5^)	STATE vs TIME
1C	65	1.33	0.73	
2C	0	–	0.5	
3C	39	0.62	0.75	
4C	54	0.6	0.58	
5C	79	3.11	0.92	
6C	64	14.56	8.12	
7C	44	1.57	1.88	
8C	12	0.12	0.39	
9C	100	0.17	–	
***Median (MAD)***	**54.0 (24.0)**	**0.98 (3.04)**	**0.74 (1.63)**	
1L	100	0.76	–	
2L	100	0.4	–	
3L	100	0.28	–	
4L	100	1.05	–	
5L	100	0.2	–	
6L	0	–	1.9	
***Median (MAD)***	**100.0 (27.8)**	**0.4 (0.3)**	**1.9 (−)**	
1R	28	0.71	1.09	
2R	75	3.06	1.07	
3R	11	0.25	3.23	
4R	57	1.33	0.35	
5R	0	–	1.85	
6R	100	1.84	–	
7R	0	–	1.06	
8R	22	0.33	0.52	
9R	100	0.28	–	
10R	100	0.31	–	
***Median (MAD)***	**42.5 (37.1)**	**0.52 (0.8)**	**1.07 (0.7)**	

State occupancies are presented in Table 3 for the case when *k* was set to the number of participants in each group. For the left FLE group, four of the six states (2, 4, 5, 6) were unique to an individual participant (5L, 6L, 1L, 4L, respectively). State 1 was mostly unique to participant 2L, but participant 3L occupied this state 19 % of the time. State 3 was identified in participant 3L and 4L. For the right FLE group, six of the 10 states (3, 4, 5, 7, 8, 9) were unique to an individual participant (4R, 8R, 5R, 2R, 1R, 3R, respectively). The other four states were identified in two to four participants. For the control group, four of the nine states (2, 3, 5, 8) were unique to an individual participant (3C, 4C, 1C, 7C, respectively). Two states (6 and 9) were unique to participant 6C, and three states (1, 4, 7) were identified in three to seven participants, with one participant occupying 100 % of their time in one of these states (2C, State 1; 8C, State 4; 9C, State 7).

**Table 3:** Somatomotor network state occupancies for participants of each group when *k* was set to the number of participants in that group (*k* = 6 for left FLE, *k* = 10 for right FLE, *k* = 9 for controls).

PARTICIPANT	STATE 1 OCCUPANCY (%)	STATE 2 OCCUPANCY (%)	STATE 3 OCCUPANCY (%)	STATE 4 OCCUPANCY (%)	STATE 5 OCCUPANCY (%)	STATE 6 OCCUPANCY (%)	STATE 7 OCCUPANCY (%)	STATE 8 OCCUPANCY (%)	STATE 9 OCCUPANCY (%)	STATE 10 OCCUPANCY (%)
**1L**	0	0	0	0	100	0				
**2L**	100	0	0	0	0	0				
**3L**	19	0	81	0	0	0				
**4L**	0	0	50	0	0	50				
**5L**	0	100	0	0	0	0				
**6L**	0	0	0	100	0	0				
**1R**	0	0	0	0	0	34	0	66	0	0
**2R**	0	29	0	0	0	0	71	0	0	0
**3R**	36	10	0	0	0	0	0	0	54	0
**4R**	0	0	64	0	0	0	0	0	0	36
**5R**	0	0	0	0	100	0	0	0	0	0
**6R**	0	0	0	0	0	0	0	0	0	100
**7R**	0	33	0	0	0	67	0	0	0	0
**8R**	0	0	0	100	0	0	0	0	0	0
**9R**	100	0	0	0	0	0	0	0	0	0
**10R**	40	60	0	0	0	0	0	0	0	0
**1C**	0	0	0	0	39	0	61	0	0	
**2C**	100	0	0	0	0	0	0	0	0	
**3C**	4	60	0	2	0	0	34	0	0	
**4C**	0	0	53	0	0	0	47	0	0	
**5C**	0	0	0	24	0	0	76	0	0	
**6C**	7	0	0	2	0	49	20	0	22	
**7C**	0	0	0	21	0	0	43	36	0	
**8C**	0	0	0	100	0	0	0	0	0	
**9C**	0	0	0	0	0	0	100	0	0	

### 3.2 Default mode network (DMN)

**Fig. 3:**
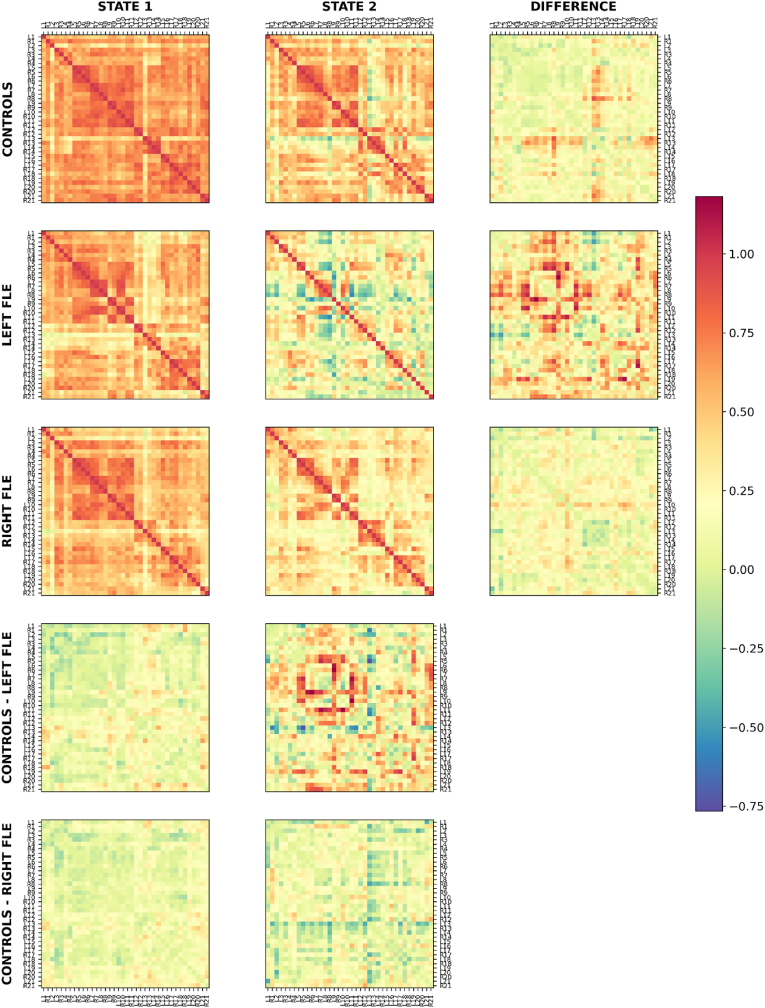
The two connectivity matrix states of the default mode network (DMN) (top left and top middle columns) identified by *k*-means clustering for control, left FLE, and right FLE participants. The top right column shows the subtraction of the two states within each group, and the bottom two rows depict the subtraction of the states between controls and each of the left FLE and right FLE groups. For controls, State 2 exhibited reduced connectivity of the medial prefrontal cortex (13, 14). State 2 for the left FLE group consisted of reduced connectivity of the ventral precuneus (5), left occipitoparietal sulcus (L9/L11) and left anterior superior frontal sulcus (L19). State 2 for the right FLE group was associated with a more overall reduction in connectivity throughout the DMN. Compared to controls, both FLE groups demonstrated lower overall connectivity, particularly in the low FC state; however, regions L13/R13 demonstrated relatively increased connectivity in both FLE groups, particularly in the left FLE group, suggesting abnormal hyperconnectivity in medial/posterior DMN regions.

**Fig. 4:**
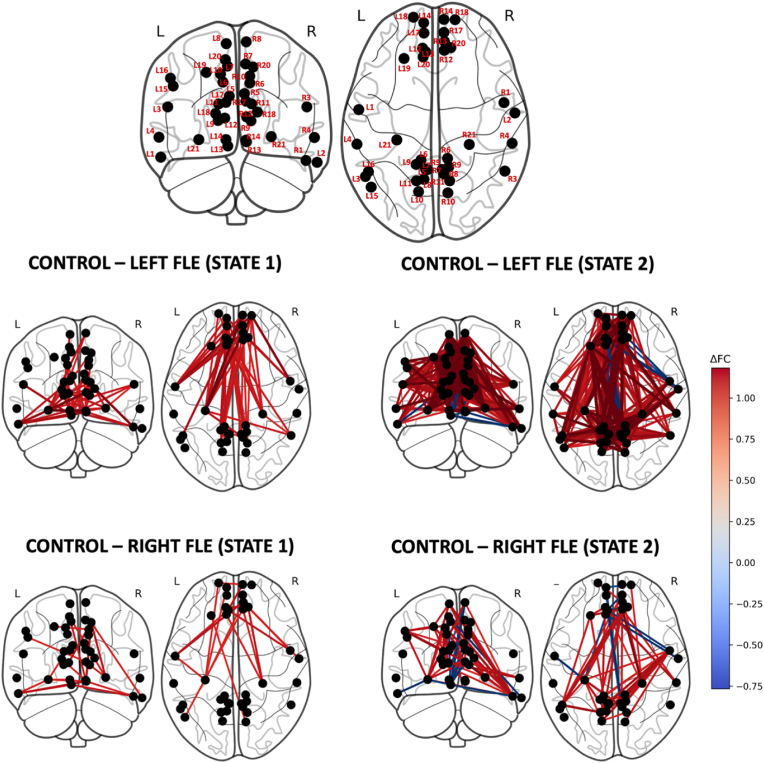
Brain models depicting the differences in connectivity of DMN brain states between controls and left FLE patients (middle row) and right FLE patients (bottom row). The left FLE group exhibited focal disruptions in ventral and lateral DMN regions, while the right FLE group exhibited more diffuse reductions. Between-group comparisons highlighted localized hyperconnectivity in posterior DMN regions (L13/R13) in both FLE groups relative to controls, shown in blue. The brain regions are labelled in the top row, as per Supplementary Table S2.

As for the SMN, the consensus amongst *k* optimization methods for the DMN for the right and left FLE groups was *k =* 2. However, unlike the SMN, no consensus was reached for the control group. Two methods (Elbow and Dunn's Score) identified 2 as the optimal value for *k*, while the other two (Silhouette and Davies-Bouldin) suggested 3 (see Supplementary Fig. 3). However, it was determined that the third state was occupied solely by a single control subject and thus not representative of the group. Therefore, to maintain consistency with the other groups and to permit between-group comparisons, *k* = 2 was chosen for controls. Fig. 3 shows the two connectivity matrix states for each group, the difference between the two states for each group, and the difference between controls and each of the left and right FLE groups for each state. Regardless of group, one state consisted of high connectivity throughout most of the DMN, and the other state consisted of lower, more variable connectivity. For the control group, State 2 was characterized by a proportionally greater reduction in connectivity between the medial prefrontal cortex (13) and the precuneus (5,7,8), posterior cingulate cortex (6), occipitoparietal sulcus (9,10,11), superior frontal gyrus (20) and hippocampus (21). State 2 for the left FLE group consisted of reduced connectivity of the ventral precuneus (5), the left occipitoparietal sulcus (L9/L11) and the left anterior superior frontal sulcus (L19). State 2 for the right FLE group was associated with a more overall reduction in connectivity throughout the DMN. The results for the DMN are also presented using brain surface plots in Fig. 4.

State occupancy, SSD, and state transition plots for all participants are provided in Table 4. For control participants, the median occupancy for State 1 was 49 %, indicating that participants occupied the two states evenly; however, three participants spent their entire time in one state only, and State 1 was not a good representation for participants 5C and 6C, as indicated by high SSD values. Except for participant 7C, all participants exhibited two or fewer transitions between states, indicating stable DMN states. For the left FLE group, five participants occupied a single state and participant L6 solely occupied a unique state, as observed previously for the SMN, indicating a lack of transitioning and highly stable states. For the right FLE group, only three participants alternated between the two states, while the remaining seven spent their entire time in only one state, reflecting a low frequency of state transitions. Whereas for the SMN, the right FLE group predominantly occupied a lower connectivity state, for the DMN, the right FLE group preferentially occupied the higher connectivity state (median occupancy of 73.5 %). As for the SMN, the procedure was repeated for the left FLE group after removing participant 6L to see if *k*-means clustering would identify states more representative of the group. The results are shown in Supplementary Fig. 4. Participants 1L and 2L occupied State 1 only, and participants 3L and 5L occupied State 2 only. Participant 4L occupied State 1 50 % of the time. State 2 was associated with decreased connectivity of the anterior ventral medial prefrontal cortex (L14/R14). Hence, *k*-means clustering identified subgroups. For the DMN, seizure burden was not significantly associated with the SSD of either State 1 (U = 33, p = 0.50) or State 2 (U = 3, p = 0.13).

**Table 4:** Default mode network state occupancy and sum of squared distances for each participant of each group (C = controls, L = left FLE, R = right FLE), as well as state versus time. Median and median absolute deviation (MAD) are reported to summarize central tendency and variability.

PARTICIPANT	STATE 1 OCCUPANCY(%)	SSD TO STATE 1 (× 10^5^)	SSD TO STATE 2 (× 10^5^)	STATE vs TIME
1C	36	41.4	65.2	
2C	27	19.4	48.8	
3C	0	–	31.7	
4C	18	21.6	78	
5C	68	315.6	150.7	
6C	84	158	30.7	
7C	49	67.8	71.4	
8C	100	76	–	
9C	100	34.6	–	
***Median (MAD)***	**49.0 (30.6)**	**54.6 (72.5)**	**65.2 (27.4)**	
1L	100	30.4	–	
2L	100	14.7	–	
3L	100	36.7	–	
4L	100	19.9	–	
5L	100	20	–	
6L	0	–	7.1	
***Median (MAD)***	**100 (27.8)**	**20.0 (7.4)**	**7.1 (−)**	
1R	20	7.5	23.6	
2R	100	15.7	–	
3R	28	23.4	61.8	
4R	100	23.3	–	
5R	100	32.2	–	
6R	47	21.9	16.2	
7R	0	–	20.9	
8R	0	–	19	
9R	100	13.1	–	
10R	100	26.9	–	
***Median (MAD)***	**73.5 (40.5)**	**22.6 (6.3)**	**20.9 (13.4)**	

State occupancies are presented in Table 5 for the case when *k* was set to the number of participants in each group. For the left FLE group, all six states were unique to an individual participant. For the right FLE group, nine of the 10 states were unique to an individual participant. The remaining state (State 1) was occupied by participant 7R 100 % of the time but was also occupied by participant 10R 27 % of the time. For the control group, seven of the nine states were unique to an individual participant (States 7 and 8 were occupied solely by participant 7C). State 1 was identified in 6 participants.

**Table 5:** Default mode network state occupancies for participants of each group when *k* was set to the number of participants in that group (*k* = 6 for left FLE, *k* = 10 for right FLE, *k* = 9 for controls).

PARTICIPANT	STATE 1 OCCUPANCY (%)	STATE 2 OCCUPANCY (%)	STATE 3 OCCUPANCY (%)	STATE 4 OCCUPANCY (%)	STATE 5 OCCUPANCY (%)	STATE 6 OCCUPANCY (%)	STATE 7 OCCUPANCY (%)	STATE 8 OCCUPANCY (%)	STATE 9 OCCUPANCY (%)	STATE 10 OCCUPANCY (%)
**1L**	0	0	0	0	100	0				
**2L**	100	0	0	0	0	0				
**3L**	19	0	81	0	0	0				
**4L**	0	0	50	0	0	50				
**5L**	0	100	0	0	0	0				
**6L**	0	0	0	100	0	0				
**1R**	0	0	0	0	0	34	0	66	0	0
**2R**	0	29	0	0	0	0	71	0	0	0
**3R**	36	10	0	0	0	0	0	0	54	0
**4R**	0	0	64	0	0	0	0	0	0	36
**5R**	0	0	0	0	100	0	0	0	0	0
**6R**	0	0	0	0	0	0	0	0	0	100
**7R**	0	33	0	0	0	67	0	0	0	0
**8R**	0	0	0	100	0	0	0	0	0	0
**9R**	100	0	0	0	0	0	0	0	0	0
**10R**	40	60	0	0	0	0	0	0	0	0
**1C**	0	0	0	0	39	0	61	0	0	
**2C**	100	0	0	0	0	0	0	0	0	
**3C**	4	60	0	2	0	0	34	0	0	
**4C**	0	0	53	0	0	0	47	0	0	
**5C**	0	0	0	24	0	0	76	0	0	
**6C**	7	0	0	2	0	49	20	0	22	
**7C**	0	0	0	21	0	0	43	36	0	
**8C**	0	0	0	100	0	0	0	0	0	
**9C**	0	0	0	0	0	0	100	0	0	

## 4 Discussion

In this study, using dynamic functional connectivity analysis combined with *k*-means clustering, we identified two states of rest for the SMN and DMN for each of healthy controls and patients with left or right FLE. In general, for FLE patients, one state was associated with high connectivity throughout the networks, whereas the second state was associated with a connectivity pattern that was attributed to an individual participant (for left FLE) or a small subgroup of participants (for right FLE and controls), rather than representative of the group. One potential interpretation of this finding is that *k*-means clustering can identify connectivity patterns unique to an individual FLE patient. This has not been demonstrated previously. This observation supports the assertion that the impact of FLE on connectivity of the SMN and DMN is unique to individual patients and involves regions within the frontal and parietal lobes. This agrees with a previous dynamic connectivity study of FLE (Klugah-Brown et al., 2019) that demonstrated decreased connectivity within frontoparietal regions in correlation with the age of onset. The identification of two dynamic states characterized by high and low connectivity within the SMN and DMN aligns with previous work demonstrating reliable separation of brain states into high- and low-connectivity regimes (F. Liu et al., 2017; Vidaurre et al., 2017), supporting the interpretability of clustering-derived connectivity states for clinical populations. More recently, despite using a different methodological approach based on dynamic effective connectivity, another study similarly identified two connectivity states within the DMN in patients with juvenile myoclonic epilepsy, one more frequent and weakly connected, the other less frequent and strongly connected (Zhang et al., 2020). These conceptually consistent findings further support our results using undirected functional connectivity.

**Extending these observations to our FLE cohort, we found** that while controls and right FLE patients showed relatively uniform connectivity reductions during the low connectivity state, left FLE patients exhibited more spatially and laterally specific disruption, i.e., reduced connectivity between lateral and medial motor regions of the SMN, and decreased connectivity of posterior and left-lateralized DMN regions. Between-group comparisons showed that both FLE groups exhibited reduced overall connectivity compared to controls, with the most prominent differences emerging during the low connectivity state (State 2). This pattern of reduced connectivity in epilepsy, particularly during states of low connectivity, has been widely reported in previous connectivity studies across various epilepsy subtypes (F. Liu et al., 2017; Pedersen et al., 2017; Morgan et al., 2015; Morgan et al., 2020; Laufs et al., 2014; Braakman et al., 2013). Notably, left FLE patients exhibited distinct alterations in the SMN, along with relative hyperconnectivity in medial/posterior DMN regions (e.g., medial prefrontal cortex). When the number of states (*k)* was increased to match the number of participants in a group, many states were identified as unique to a single participant, particularly within the left FLE group, highlighting an individualized nature of functional connectivity patterns in epilepsy and emphasizing the potential value of subject-specific analyses when investigating dynamic brain states.

Additionally, state occupancy analysis revealed that left FLE patients spent nearly all of their time in the high connectivity state, in contrast to prior findings in generalized epilepsy patients, where weakly connected states predominated (Li et al., 2024). Our findings, however, align with prior research suggesting that left FLE patients may undergo network-level adaptations to preserve cognitive function. Persistent high connectivity states, particularly in the DMN, may reflect compensatory or maladaptive reorganization, consistent with studies linking altered DMN connectivity to cognitive deficits (Fahoum et al., 2012; McCormick et al., 2013; W. Liu et al., 2021; Haneef et al., 2012; Yang et al., 2021). The functional significance of the identified states remains unclear: high connectivity may reflect compensatory integration, but in some cases, particularly for left FLE, it may also signal maladaptive hypersynchrony or reduced network flexibility (Zhang et al., 2010b; Shine et al., 2016). In the SMN, the predominance of a high connectivity state may relate to motor planning deficits and, in the DMN, to pathological hyperconnectivity that disrupts cognitive processes (Douw et al., 2015; Shine et al., 2017). Since only two stable states with few transitions were identified for both the SMN and DMN, occurrence frequency was not analyzed.

While a significant association between DMN connectivity and disease burden has been demonstrated in previous studies (Fahoum et al., 2012; Liao et al., 2010), a similar association was not observed in the present study for brain states. Given the limited number of patients, our study may not have been sufficiently powered to elucidate a direct relationship between brain state and seizure burden. Future studies of seizure burden with larger cohorts are thus warranted.

A major limitation of the present study is the small group sizes due to the challenges associated with recruiting FLE patients. Nevertheless, our cohort, consisting of FLE patients that our lab previously investigated with a more conventional resting-state fMRI analysis approach (Woodward et al., 2014), and additional patients, provided a valuable opportunity to assess the feasibility and clinical relevance of our proposed dynamic connectivity analysis framework. Our findings demonstrate the utility of our approach and help inform and validate its application in a larger cohort of patients in future studies. Our study did not examine the effects of the location of seizure focus or symptoms due to limited group sizes. To this end, larger group sizes may provide an opportunity to determine connectivity patterns that are more common amongst FLE patients with similar aetiologies, seizure burdens, or behavioural symptoms. In addition, the participants in our study varied in age and age of seizure onset. These factors could also be examined with larger group sizes. Furthermore, all patients were taking one or more antiseizure medications, which could potentially affect connectivity measurements and thus warrants further investigation. Previous studies have reported mixed effects of these medications on fMRI metrics (Szaflarski and Allendorfer, 2012; Pressl et al., 2019), and thus their impact on resting-state connectivity remains unclear. Certain agents, including carbamazepine and valproate, have been associated with altered intrinsic network coherence, particularly within the DMN (Tracy et al., 2014; Xiao et al., 2019). Future studies involving medication-controlled or drug-naïve cohorts will be critical to disentangle treatment effects from epilepsy-related disruptions in brain connectivity.

Clinically, our findings may have implications for the management and treatment of FLE patients. The ability to identify unique connectivity patterns in individual patients could pave the way for personalized diagnostic and treatment strategies in the future. For instance, interventions could be tailored based on specific connectivity alterations observed in each patient, potentially improving treatment efficacy and patient outcomes. Additionally, understanding the unique connectivity patterns associated with different seizure burdens, seizure foci, and behavioural symptoms could lead to more precise and effective therapeutic approaches.

In summary, dynamic connectivity analysis combined with *k*-means clustering has the ability to determine the impact of FLE on the connectivity of the SMN and DMN at the individual FLE patient level. With further refinement, our approach may have the potential to contribute to the clinical management of FLE patients, as it may assist in the development of personalized diagnostic and treatment strategies.

## CRediT authorship contribution statement

**Tahereh Rashnavadi:** Writing – review & editing, Writing – original draft, Visualization, Validation, Software, Methodology, Investigation, Formal analysis, Data curation, Conceptualization. **Raphael F. Casseb:** Software. **Kristine E. Woodward:** Data curation. **Paolo Federico:** Writing – review & editing, Supervision, Resources, Funding acquisition. **Bradley Goodyear:** Writing – review & editing, Validation, Supervision, Resources, Project administration, Methodology, Conceptualization.

## Funding

This work was supported by the Canadian Institutes of Health Research (CIHR), Grant Number MOP-136839.

## Declaration of competing interest

The authors declare that they have no known competing financial interests or personal relationships that could have appeared to influence the work reported in this paper.

## Data Availability

Anonymized data that support the findings of this study are available upon request from the corresponding author and approval by the Conjoint Health Research Ethics Board of the University of Calgary. The data are not publicly available as they contain patient identifying information that could compromise the privacy of research participants.
