# Supplementary material for: Motor and default mode network states of rest in frontal lobe epilepsy
Source: Neuroimage Rep. 2025 Jun 28;5(3):100278. doi: 10.1016/j.ynirp.2025.100278 (PMC12489773; doi:10.1016/j.ynirp.2025.100278)
Supplement: Multimedia component 1 [file mmc1.pdf]

SUPPLEMENTARY TABLES

Table S1. Regions of interest (ROIs) of the somatomotor network (SMN). Each ROI is located in the left and right hemispheres, resulting in 12 ROIs.

<table>
<tr><td colspan="3">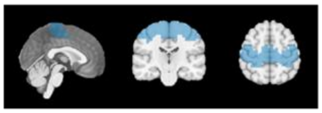</td></tr>
<tr><th>ROI</th><th>Brain Region</th><th>Brain Region Location</th></tr>
<tr><td>1</td><td>Ventrolateral</td><td>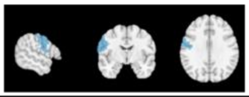</td></tr>
<tr><td>2</td><td>Dorsolateral</td><td>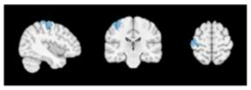</td></tr>
<tr><td>3</td><td>Lateral</td><td>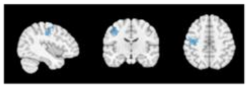</td></tr>
<tr><td>4</td><td>Anteromedial</td><td>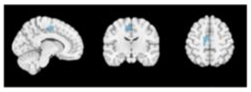</td></tr>
<tr><td>5</td><td>Medial</td><td>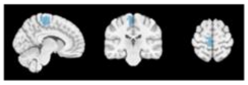</td></tr>
<tr><td>6</td><td>Mediolateral</td><td>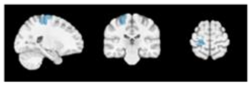</td></tr>
</table>

Table S2. Regions of interest (ROIs) of the default mode network (DMN). With the exception of ROIs 15, 16, and 19 (which only have presentations in the left hemisphere) and ROI2 (which appears bilaterally (despite being labelled as L2)), each ROI is located in the left and right hemispheres, resulting in 38 ROIS.

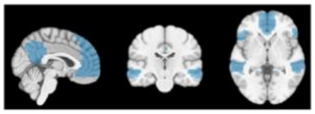

ROI	Brain Region	Brain Region Location		ROI	Brain Region	Brain Region Location
1	Anterior Middle Temporal Gyrus	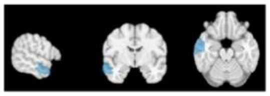		12	Perigenual Anterior Cingulate Cortex	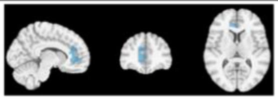
2	Ventral Anterior Middle Temporal Gyrus	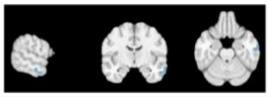		13	Posterior Ventral Medial Prefrontal Cortex	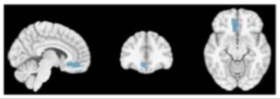
3	Angular Gyrus	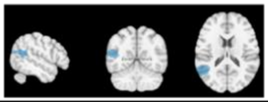		14	Anterior Ventral Medial Prefrontal Cortex	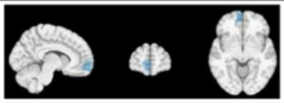
4	Posterior Middle Temporal Gyrus	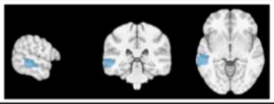		15	Inferior Parietal Lobule	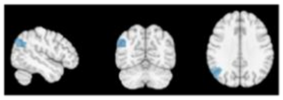
5	Ventral Precuneus	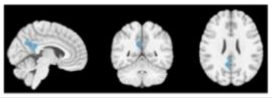		16	Inferior Parietal Lobule	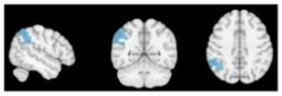
6	Posterior Cingulate Cortex	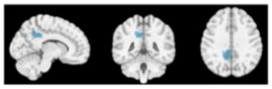		17	Anterocaudal Dorsomedial Prefrontal Cortex	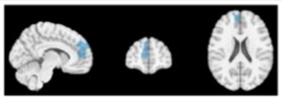
7	Inferior Dorsal Precuneus	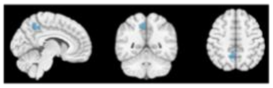		18	Anterorostral Dorsomedial Prefrontal Cortex	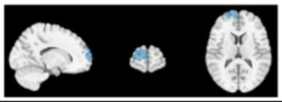
8	Superior Dorsal Precuneus	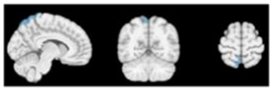		19	Anterior Superior Frontal Sulcus	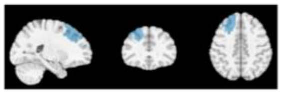
9	Ventral Occipitoparietal Sulcus	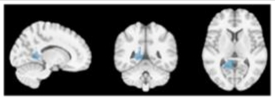		20	Anterodorsal Superior Frontal Gyrus	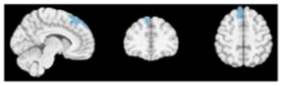
10	Dorsal Occipitoparietal Sulcus	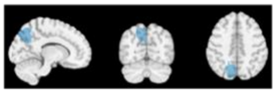		21	Hippocampus	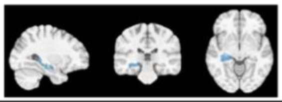
11	Occipitoparietal Sulcus	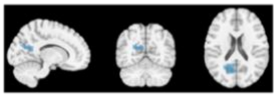				

Figure S1. The results for k optimization using four methods of elbow, silhouette, Davies-Bouldin, and Dunn's score of the SMN for each group. Regardless of group, the consensus across methods was $k_{opt} = 2$.

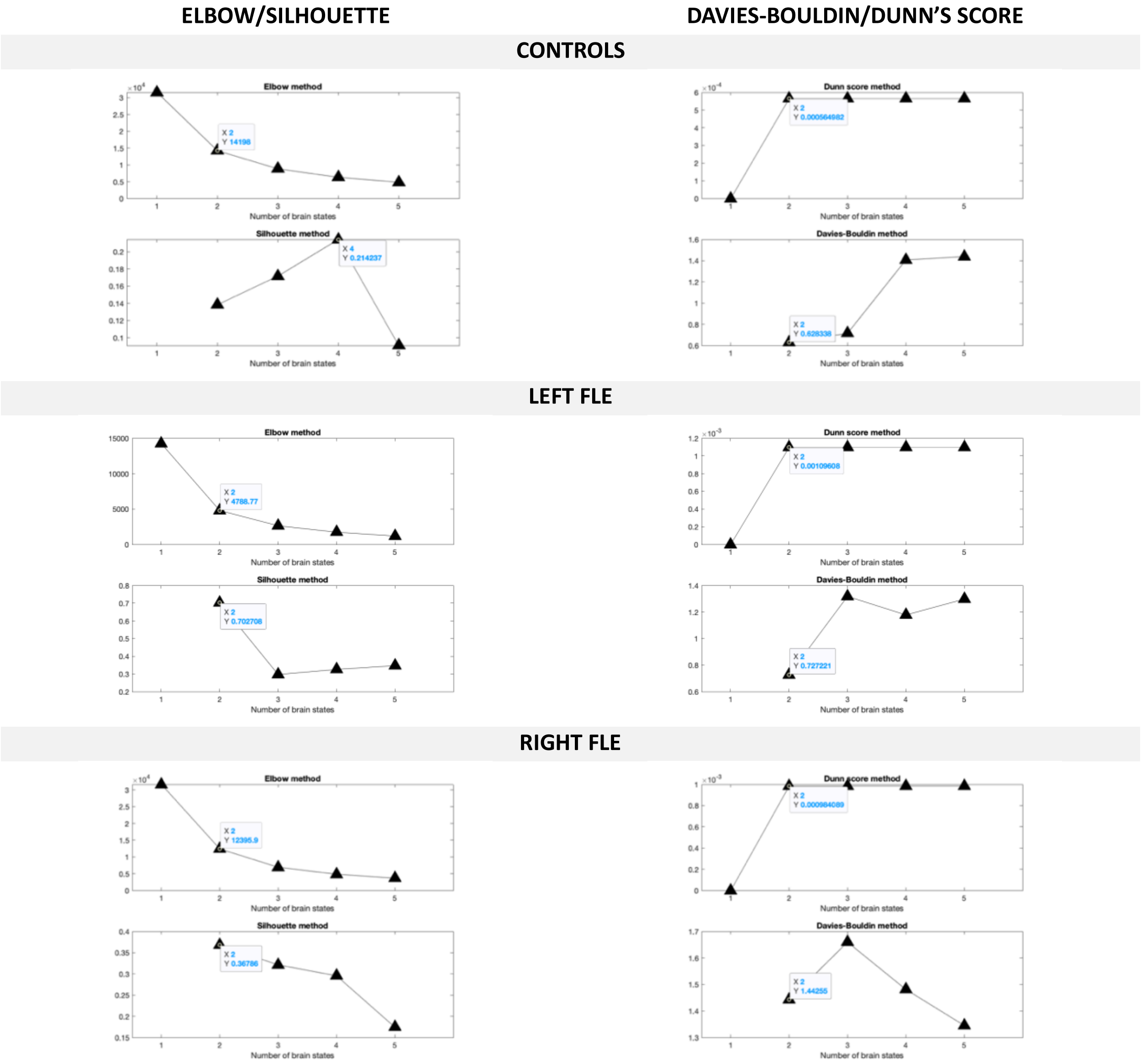

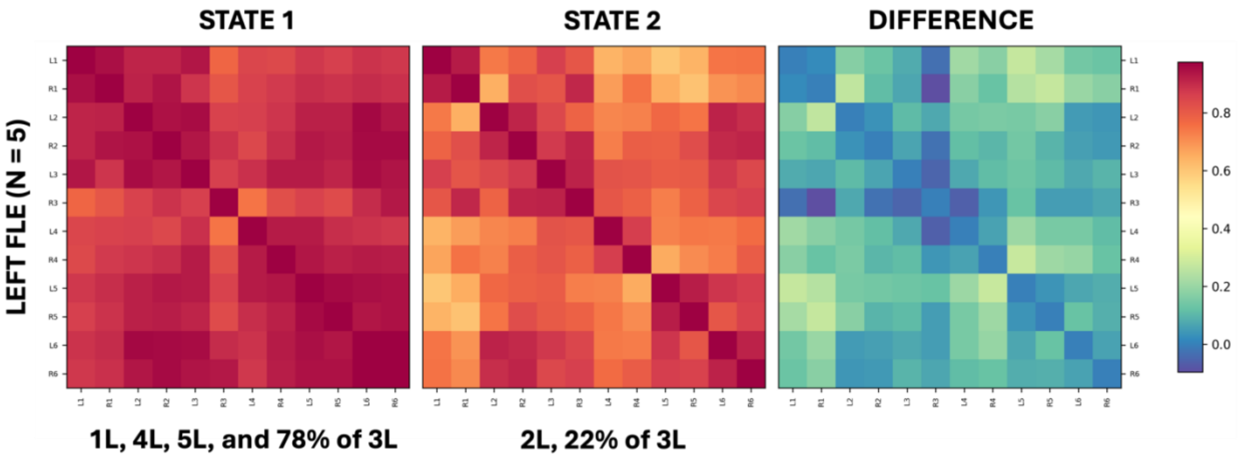

Figure S2. The two connectivity matrix states of the SMN (left and middle columns) identified by *k*-means clustering after removing participant 6L. The right column shows the subtraction of the two states. Participants 1L, 4L and 5L occupied State 1 only, and participant 2L occupied State 2 only. Participant 3L occupied State 1 78% of the time. State 2 was associated with decreased connectivity between the ventrolateral (L1/R1), anteromedial (L4/R4) and medial regions (L5/R5) of SMN in both hemispheres.

Figure S3. The results for k optimization using four methods of Elbow, Silhouette, Davies-Bouldin, and Dunn's score of the DMN for each participant group. For the right and left FLE groups, the consensus across methods was $k_{opt} = 2$, while for control group, both k values of 2 and 3 were suggested as the optimal.

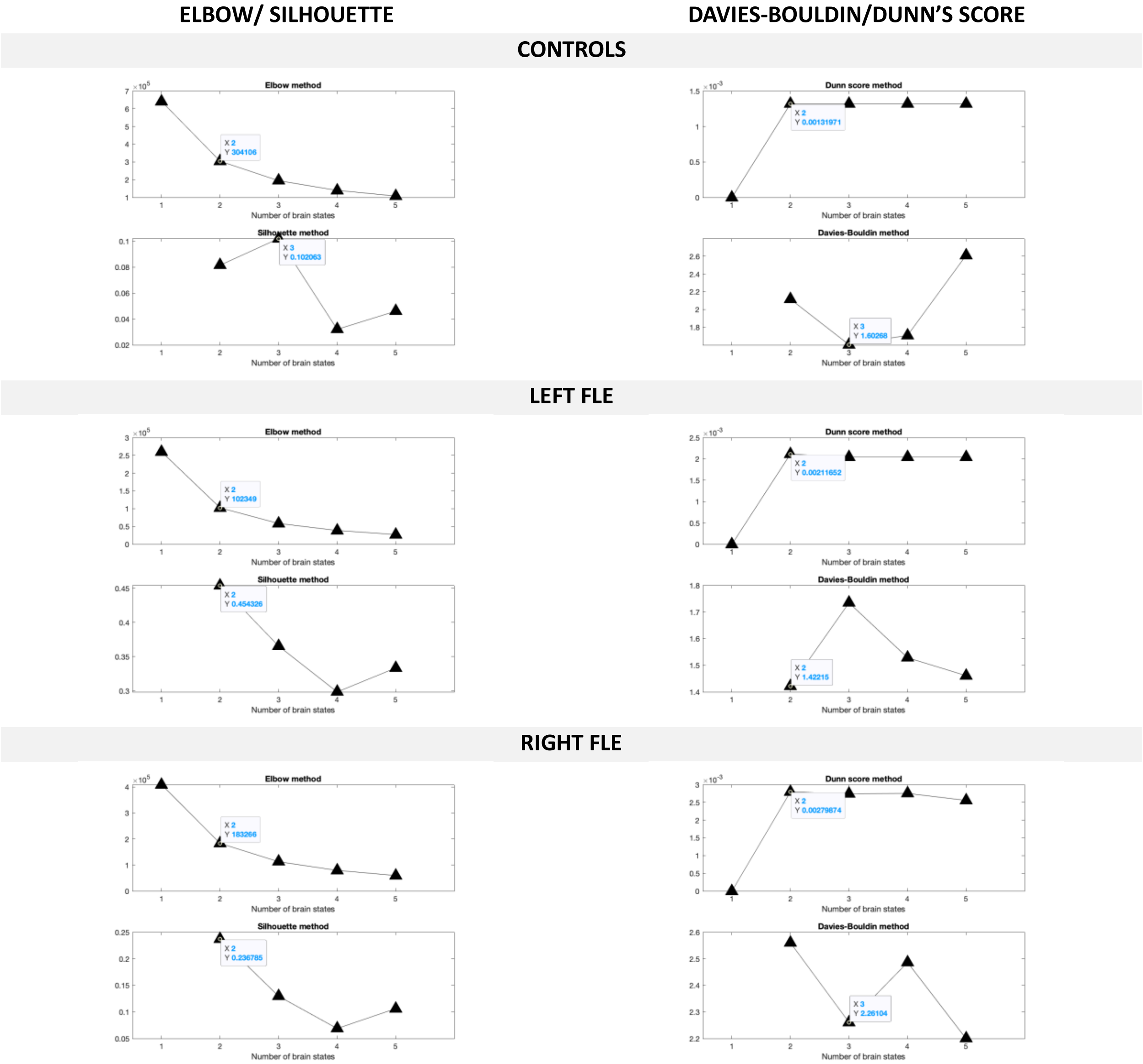

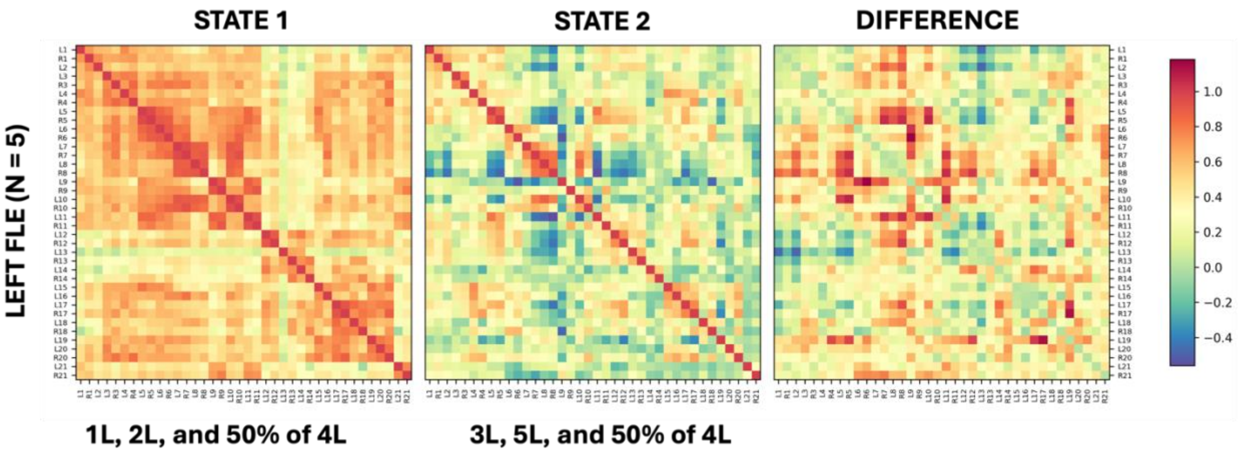

Figure S4. The two connectivity matrix states of the DMN (left and middle columns) identified by *k*-means clustering after removing participant 6L. The right column shows the subtraction of the two states. Participants 1L and 2L occupied State 1 only, and participants 3L and 5L occupied State 2 only. Participant 4L occupied State 1 50% of the time. State 2 was associated with decreased connectivity of the anterior ventral medial prefrontal cortex (L14/R14).
